# Liquid Regions of Lanthanum-Bearing Aluminosilicates

**DOI:** 10.3390/ma13020450

**Published:** 2020-01-17

**Authors:** Yandong Li, Tongsheng Zhang, Yefeng Feng, Chengjun Liu, Maofa Jiang

**Affiliations:** 1 Key Laboratory of Extraordinary Bond Engineering and Advanced Materials Technology (EBEAM), Yangtze Normal University, Chongqing 408100, China; andyydlee@gmail.com (Y.L.); feng_ye_feng@126.com (Y.F.); 2 Key Laboratory of Ecological Utilization of Multi-Metallic Mineral of Education Ministry, Northeastern University, Shenyang 110819, China; liucj@smm.neu.edu.cn (C.L.); jiangmf@smm.neu.edu.cn (M.J.); 3 National Center for International Research of Clean Metallurgy, Central South University, Changsha 410083, China

**Keywords:** rare earth, CALPHD (Computer Coupling of Phase Diagrams and Thermochemistry), glass, phase diagram

## Abstract

The Al_2_O_3_-SiO_2_, La_2_O_3_-Al_2_O_3_, and La_2_O_3_-SiO_2_ binary phase diagrams were estimated by Redlich–Kister expression. La_4.67_Si_3_O_13_ (=La_4.67_(SiO_4_)_3_O) was introduced to improve the existing phase diagrams. The Al_2_O_3_-SiO_2_-La_2_O_3_ ternary phase diagram extrapolated by Kohler method was optimized. Then, the liquidus of Al_2_O_3_-SiO_2_-La_2_O_3_ system at 1600 °C was compared with Al_2_O_3_-SiO_2_-RE_2_O_3_ (RE = Rare Earth Elements) systems and experimental results in other literature. The high temperature experiments were conducted in the tube furnace at 1500 °C. Then the field emission scanning electron microscope (FE-SEM), energy dispersive spectrometer (EDS), and X-ray diffraction (XRD) were employed to verify the calculated liquid region and precipitates phase at 1500 °C. Moreover, the liquidus of binary systems were compared with FactSage results and experiments. The optimized ternary phase diagram shows the relatively reliable region of liquid phase, and it is significant to the seal glass of solid oxide fuel cells and other fields being related to RE containing silicates.

## 1. Introduction

Aluminosilicate glasses containing rare-earth (RE) elements have been suggested for various applications owing to its favorable chemical, mechanical and optical properties and so on [1,2,3]. They can constitute host materials in optical devices and are proposed for radioactive waste-storage [4]. Moreover, RE-bearing silicate glass is the favorable material to be the seal glass for solid oxide fuel cells (SOFC) owing to the critical thermal properties [5]. RE_2_O_3_-Al_2_O_3_-SiO_2_ systems reveal relatively wide glass formation regions when prepared from melts of given compositions between approximately 1500 °C and 2000 °C [6,7]. To well understand the glass formation region and the associated microstructure and reaction thermodynamics of the mentioned system, an explicit phase diagram of RE_2_O_3_-Al_2_O_3_-SiO_2_ systems can provide precise information of these. However, few of experimental data of the phase diagrams are available to present the liquid region (homogeneous glass formation region) in RE_2_O_3_-Al_2_O_3_-SiO_2_ systems (RE elements are mainly La [8], Y [9,10,11,12] and Gd [11,12]) and the nonstoichiometric phases in each binary system. Mullite and spinel are always formed simultaneously or in order in the Al_2_O_3_-SiO_2_ system [13]. The oxyapatite phase is found in the La_2_O_3_-SiO_2_ system [14]. The RE_2_O_3_-Al_2_O_3_-SiO_2_ systems (RE = Nd, Sm, Gd and La) were computed by simplified thermodynamic properties associating with symmetric extrapolated expression [15,16,17]. Nevertheless, the liquid region of La_2_O_3_-Al_2_O_3_-SiO_2_ system is much different from the experimental results given by Iftekhar [18]. Only limited publications can be consulted about the isothermal section information of the La_2_O_3_-Al_2_O_3_-SiO_2_ system diagram, not to mention the projection map. The phase diagram of La_2_O_3_-Al_2_O_3_-SiO_2_ system cannot be worked out by any thermodynamic software by now as a result of lacking parameters of mixed solution. Therefore, the primary task of the present work is to determine the liquid region and precipitates at high temperature (≥1500 °C) of possible glass formation, whereas the solid solutions found at 1300 °C by Mazza [8] was not considered in the present work because the temperature is low and there is no homogeneous glass formation.

A plenty of optimized systematic phase diagrams have been worked out by Computer Coupling of Phase Diagrams and Thermochemistry (CALPHAD) technique based on various solution models, which are released to describe the solution phase, such as regular solution model, sublattice model and quasi-chemical model and so on. In addition, a variety of empirical or mathematical expressions, such as Margules, Redlich–Kister, and Bale–Pletion expressions, are generated during the process of development. In the present work, the Redlich–Kister [19] expression based on the subregular solution model was adopted to calculate the Al_2_O_3_-SiO_2_, A_2_O_3_-La_2_O_3_, and La_2_O_3_-SiO_2_ binary systems, and the La_2_O_3_-Al_2_O_3_-SiO_2_ ternary system was extrapolated by Kohler method [20]. Then the optimized ternary system phase diagram was critically assessed by comparing with existing experimental results. The obtained ternary phase diagram is providing a theoretical basis for the glass production process and other fields, such as metallurgical flux preparation and green comprehensive utilization of complex cogenetic ore containing rare earth elements.

## 2. Calculation Method and Experimental Procedure

The phase boundary lines in phase diagrams are definitely drawn by connecting a series of equilibrium points at the given temperature and pressure. Every equilibrium point is carefully calculated based on the total Gibbs principle of minimum free energy under some constraints. The totally absolute Gibbs free energy of mixed system can be written as

(1) $$G=G^{ref}+G_{mix}^{id}+G^{E}=\underset{i}{\sum }x_{i}G_{i}^{0}+\mathrm{RT}\underset{i}{\sum }x_{i}\ln x_{i}+G^{E}$$

where *x_i_* is the mole fraction of component *i*; $G_{i}^{0}$ is the standard Gibbs free energy of pure substance *i*, J·mol^−1^; *R* is the gas constant, 8.314 J mol^−1^·K^−1^; *T* is the temperature, Kelvin; and *G*^E^ is excess Gibbs free energy, J·mol^−1^. 

The $G_{i}^{0}$ can be derived by Gibbs–Helmholtz expression, *G* = *H* − *TS*, from essential thermodynamic properties, such as standard enthalpy, standard entropy and isobaric heat capacity. The referable data mentioned above related to the base members in this work are listed in Table 1. For some substances such as LaAlO_3_ of which applicable temperature is out of the scope for direct calculation, the extrapolation based on the *C_p_* expression is adopted in the current optimization process.

*G^E^* in Equation (1) is the most important parameter to represent the interaction of components except for entropy value increase contributed by ideal mixture. In the present work, it can be obtained in the binary system by the Redlich–Kister expression as follows,

(2) $$\begin{array}{ll} G^{E} & =x_{1}x_{2}\overset{n}{\underset{j=0}{\sum }}L_{j}{\left(x_{1}-x_{2}\right)}^{j}\\ & \;\;\;\;\;\;=x_{1}x_{2}L_{0}+x_{1}x_{2}L_{1}\left(x_{1}-x_{2}\right)+x_{1}x_{2}L_{2}{\left(x_{1}-x_{2}\right)}^{2}+\cdots \end{array}$$

where *L_j_* (*j* = 0, 1, 2, …) is the interaction energy or interaction parameter between two end members. It can be written in the general form of Gibbs free energy as follows,

(3) $$L=a+bT+cT\ln\left(T\right)+dT^{2}+eT^{3}+fT^{-1}$$

where *a*, *b*, *c*, *d*, *e*, and *f* are the constant parameters usually obtained through empirical or semi-empirical method. Only the first two parameters *a* and *b* are considered and optimized later in this work as the deviation is acceptable in this situation. 

The commercial software FactSage 7.1 which is popular in phase diagram optimization by CALPHAD technique was applied to conduct the optimization process and also used to draw the final phase diagrams according to the user-define database. All the thermodynamic properties in Table 1 were included in the user-define database. The data of solid solution phase “mullite” was chosen from the FToxid database which is strong in the calculations of mixed oxides systems. These existing experimental data in the available literature, such as liquidus temperature and activity, were adopted to assess the calculation results in the binary phase diagrams. The modules, Solution, Equilib, Phase Diagram, and OptiSage, were employed to achieve the parameters optimization and phase diagrams calculation. Then, the calculated data points were export to the software Origin 8.5 to draw the phase diagram comparing with the reference data points or experimental results. Moreover, the optimized binary phase diagrams Al_2_O_3_-SiO_2_ and La_2_O_3_-Al_2_O_3_ systems were compared with the results computed by FactSage 7.1 where the FToxid database was employed. And the activity curves of CaO and Al_2_O_3_ in the Al_2_O_3_-SiO_2_ system were compared with the limited experimental points at 1877 °C (2150 K).

After the *G*^E^ values of binary phase diagrams were obtained, the extrapolation portion of La_2_O_3_-Al_2_O_3_-SiO_2_ ternary phase diagram was derived by Kohler method. Considering that the results calculated by extrapolation methods are not accurate enough, an additional *G*^E^ parameter optimized from the ternary eutectic temperature of La_2_O_3_-Al_2_O_3_-SiO_2_ system was introduced to correct the deviation.

**Figure 1:**
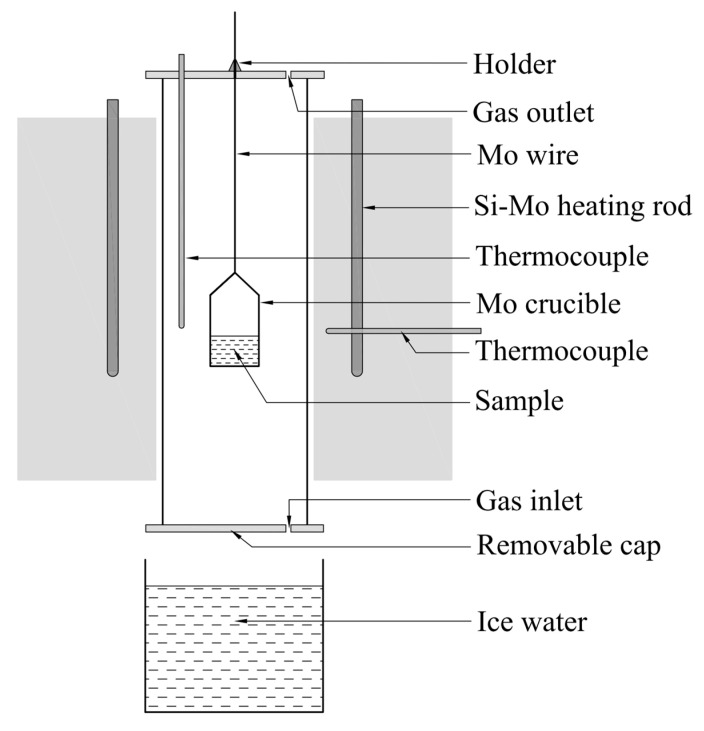
Schematic diagram of high temperature furnace.

When the ternary phase diagram of La_2_O_3_-Al_2_O_3_-SiO_2_ system were worked out, the liquid region of the system at 1600 °C was compared with the experimental results in published references. At the same time, validation experiments of liquid region at 1500 °C were carried out in a tube furnace heated by MoSi_2_ rods, the schematic of which is shown as Figure 1. 

The Al_2_O_3_, SiO_2_, and La_2_O_3_ powders with the purity of 4 N supplied by Sinopharm Chemical Reagent Company were pretreated in the Muffle furnace at 1000 °C for 2 h to remove moisture, impurity and volatiles. Then the material powders were grinded and sieved. The particles with the size less than 74 μm were intensively mixed in the agate mortar at the given mole ratios (as the 18 experimental points marked in the figures later). One gram of individual sample was taken from the evenly mixed powders and then transferred into a Mo crucible with the inner diameter of 1 cm and wall thickness of 1 mm. The powdered sample in the Mo crucible was compacted by the glass road and then pricked with stainless steel needle to make holes to avoid eruption during the heating process. Afterwards, the Mo crucible hung by the Mo wire was placed into a tube furnace at ambient temperature under argon atmosphere at a flow rate of 5.0 L·min^−1^. Then, the furnace was heated up and held at 1600 °C for 1 h to pre-melting and to ensure the totally uniformity of the sample at good flow conditions. Thereafter, the furnace temperature was slowly decreased to 1500 °C and held for 2 h. Subsequently, the holder at the top side of furnace was loosened and the sample rapidly fall under gravity to be quenched into ice water from 1500 °C to 0 °C through the bottom side of furnace as shown in Figure 1. The obtained sample was mounted in the ethoxyline resin and polished by various scale of abrasive paper and polishing cloth in order under the aid of grinding paste SiC. Gold powder was sprayed on the polished surface of sample to ensure the electrical conductivity before the following analysis. Finally, the FE-SEM with EDS and XRD were used to determine the morphology, chemical composition and mineral phase of the quenched sample.

## 3. Results and Discussion

### 3.1. Binary Phase Diagram of Al_2_O_3_-SiO_2_-La_2_O_3_ System

**Figure 2:**
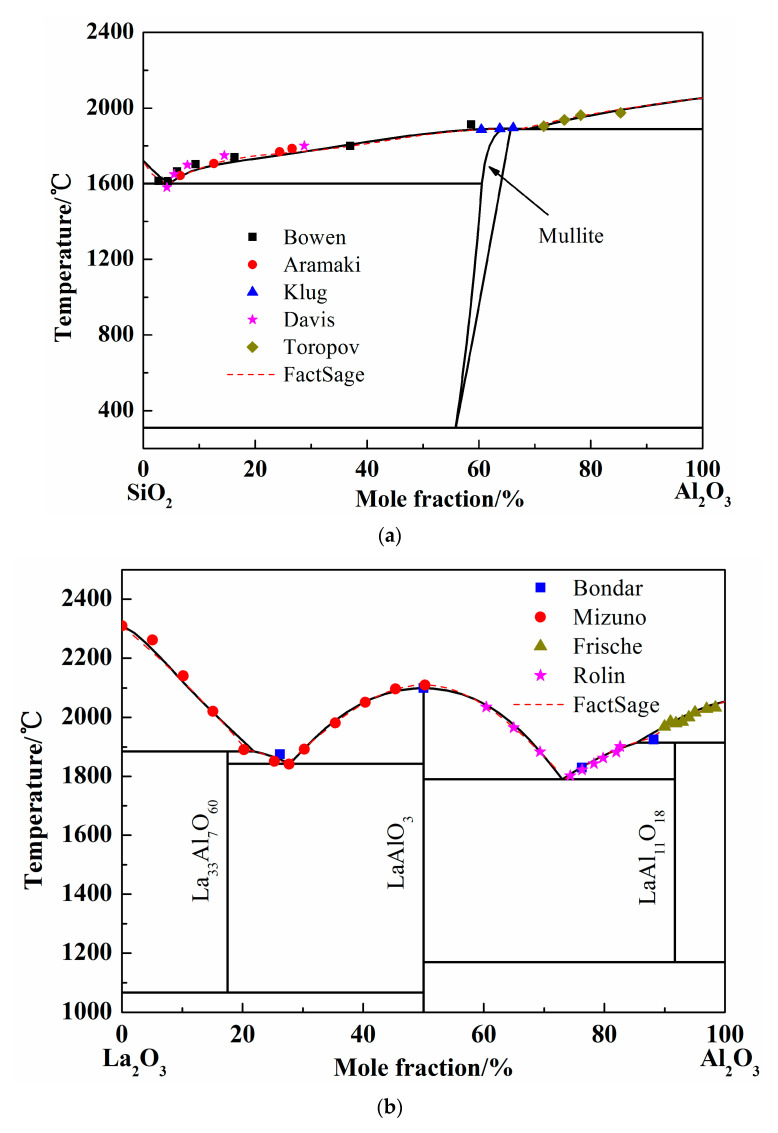

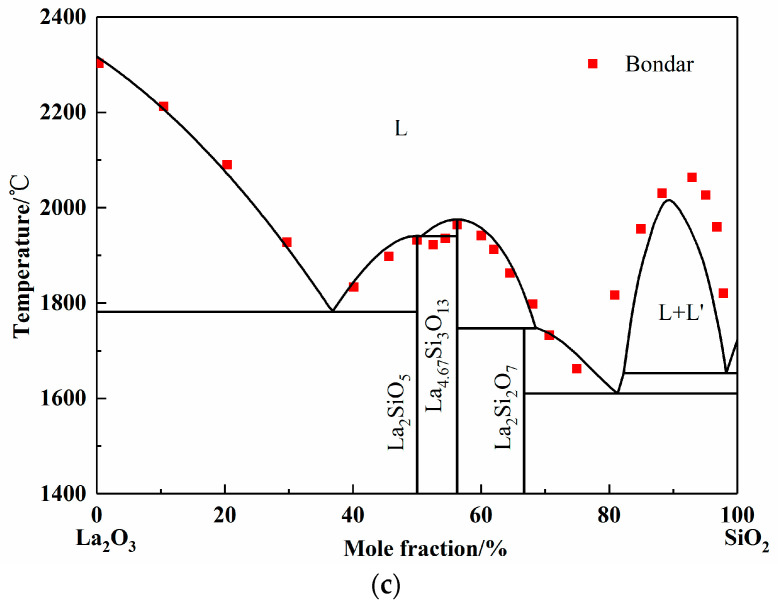
Calculated phase diagrams of binary systems. (**a**) Phase diagram of SiO_2_-Al_2_O_3_ system; (**b**) Phase diagram of La_2_O_3_-Al_2_O_3_ system; (**c**) Phase diagram of La_2_O_3_-SiO_2_ system.

When the Redlich–Kister expression based on subregular solution model and Kohler method were employed to optimize all the binary systems, the related interaction parameters were worked out and are listed in Table 2 and all the binary phase diagrams are present in Figure 2.

#### 3.1.1. The SiO_2_-Al_2_O_3_ System

To study the stable phases in the SiO_2_-Al_2_O_3_ system, various models were introduced and the “mullite” region was considered as solution phase or pure substance in previous researches. In 1979, the SiO_2_-Al_2_O_3_ phase diagram was calculated by DÖrner using regular solution model [28]. However, the liquidus was different from the available experimental results. Thereafter, the Gibbs free energy of mixing liquid was introduced using an associated solution model by Ball in 1993 [29]. The mullite region is in good agreement with the experimental results. At the same time, Eriksson conducted the calculation using modified quasi-chemical model [30] and the results were acceptable. In 2005, the two-sublattice model was adopted to calculate the SiO_2_-Al_2_O_3_ system and the optimized results coincide well with the experimental data. Besides, when the mullite was considered as pure substance, the calculated liquidus of Al_2_O_3_-SiO_2_ system showed more accuracy [15,31]. In present work, the mullite data form FactSage database was introduced and the solution phase was calculated by Redlich–Kister expression. The calculated SiO_2_-Al_2_O_3_ phase diagram was shown as Figure 2a [32,33,34,35,36]. The dash line in Figure 2a is the liquidus worked out by FactSage 7.1 when the solution phase was chosen form FToxide database. It is obvious that the liquid region calculated by Redlich–Kister expression is almost the same as the FactSage result. In addition, the experimental points employed conformably meet the calculated liquidus. 

**Figure 3:**
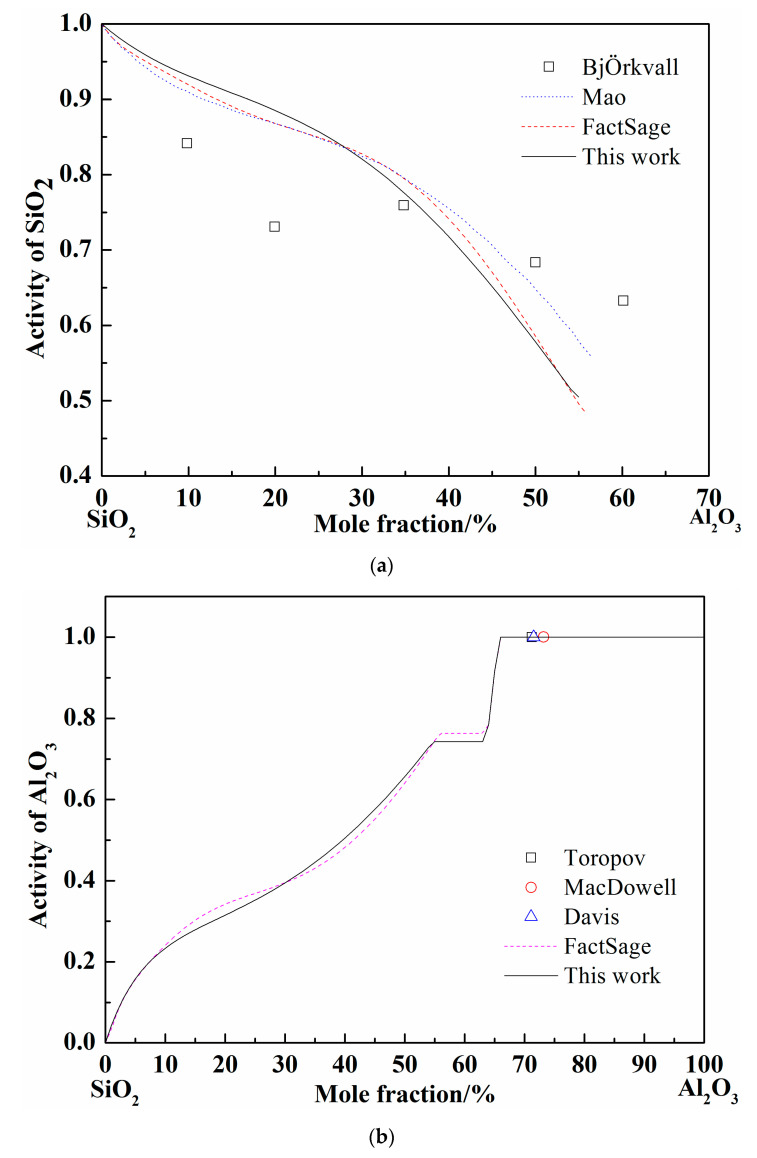
Calculated activity curves in the SiO_2_-Al_2_O_3_ system at 1877 °C. (**a**) The SiO_2_ activity in SiO_2_-Al_2_O_3_ system; (**b**) The Al_2_O_3_ activity in SiO_2_-Al_2_O_3_ system.

When the pure liquid phase was chosen as the standard state, the activity of SiO_2_ was calculated and compared with the FactSage and others’ results at 1877 °C, as shown in Figure 3a [37,38]. The activity of SiO_2_ worked out by different calculation (Mao, FactSage, and present work) adopted different models are nearly coincident. The calculated curves show the same tendency with the experimental results by BjÖkvall et al. Figure 3b shows the calculation results of Al_2_O_3_ activity at 1877 °C [36,37,39], when the pure solid phase was chosen as the standard state. The experimental points by Toropov et al. show the pure solid Al_2_O_3_ formation site as the Al_2_O_3_ content increase at 1877 °C and the Al_2_O_3_ activity is definitely 1. The variation trend of Al_2_O_3_ activity calculated by FactSage and in present work are consistent and the activity value reaches 1 at the same point (66% of Al_2_O_3_ in mole fraction). Generally, the optimized SiO_2_-Al_2_O_3_ system by Redlich–Kister expression is accurate according to the above comparison. 

#### 3.1.2. The La_2_O_3_-Al_2_O_3_ System

Only a few of experimental studies and calculations on the phase diagram La_2_O_3_-Al_2_O_3_ system are published so far. The La_2_O_3_-Al_2_O_3_ phase diagram was calculated using quasi-chemical model by Wu in 1992 [20], and using Redlich–Kister expression by Li [15]. However, the fusion enthalpy and entropy in the work of Li et al. are simplified to temperature-independent constants. The more precise thermodynamic properties should be introduced, and the La_2_O_3_-Al_2_O_3_ system was calculated by Redlich–Kister expression. The calculation results and the experimental points in the present work are shown in Figure 2b [40,41,42,43]. The FactSage results and all the experimental points by former scholars were almost coincided with the current calculated liquidus, which indicates the reliability of optimized binary phase diagram of La_2_O_3_-Al_2_O_3_ system. 

#### 3.1.3. The La_2_O_3_-SiO_2_ System

The La_2_O_3_-SiO_2_ system is rarely studied by experiment or simulation, and different opinions on the intermediate phases have always been there. In 1961, Toropov released the La_2_O_3_-SiO_2_ phase diagram, and the intermediate compounds are La_2_Si_2_O_7_, La_2_SiO_5_, and La_4_Si_3_O_12_. In 1982, Bondar, from the same research group as Toropov, modified the La_4_Si_3_O_12_ phase to La_4.67_Si_3_O_13_. Li finished the calculated La_2_O_3_-SiO_2_ system employing simplified thermodynamic properties in 1999 [11]. However, the adoptive compound was La_4_Si_3_O_12_. Kim only calculated the two-liquid region using the Redlich–Kister expression [44]. However, the number and values of parameters given by Li and Kim are completely different. In this work, La_2_Si_2_O_7_, La_2_SiO_5_, and La_4.67_Si_3_O_13_ were chosen as the intermediate compounds, which were found in the equilibrium experimental results, as shown in Figure 5 later. Since the available experimental points are from Toropov and Bondar, the interaction energy of solution phase and the derived thermodynamic parameters of silicates were optimized. The calculated phase diagram is presented in Figure 2c [45,46]. It can be seen that most of the experiment points have good agreement with the calculation results.

### 3.2. Ternary Phase Diagram of Al_2_O_3_-SiO_2_-La_2_O_3_ System

After all the binary phase diagrams of Al_2_O_3_-SiO_2_, La_2_O_3_-Al_2_O_3_, and La_2_O_3_-SiO_2_ systems were worked out, the thermodynamic property of Al_2_O_3_-SiO_2_-La_2_O_3_ system was extrapolated by Kohler method. In 1999, Li published this ternary phase diagram when the ternary interaction coefficients of solution phases were set to zero [11]. Consequently, the calculated results were distinct from others studies. In the present work, the ternary coefficients were calculated through references to the ternary eutectic temperature 1380 °C [7] and the Al_2_O_3_-Ce_2_Si_2_O_7_ system [47]. The additional *G*^E^ optimized from the ternary eutectic temperature of Al_2_O_3_-SiO_2_-La_2_O_3_ system is $-450,000x_{{\mathrm{La}}_{2}O_{3}}x_{{\mathrm{Al}}_{2}O_{3}}x_{{\mathrm{SiO}}_{2}}^{2}$ .

**Figure 4:**
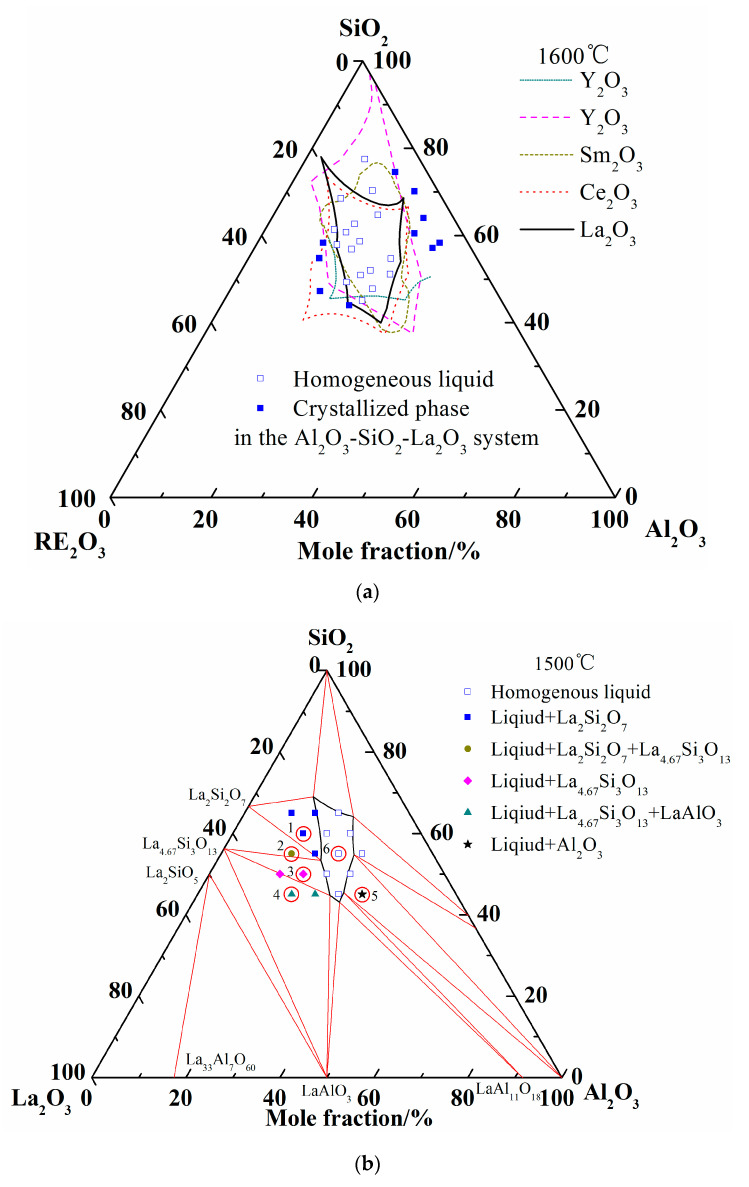
Comparison of liquid region of ternary phase diagrams. (**a**) Liquid regions of ternary phase diagrams at 1600 °C; (**b**) Liquid region and experimental points at 1500 °C.

Abundant experimental results about the glass formation region at 1600 °C from publications are available, so no more experiments were conducted in the present work. Considering the analogous chemical and physical properties of rare earth elements, the liquidus of Al_2_O_3_-SiO_2_-La_2_O_3_, Al_2_O_3_-SiO_2_-Sm_2_O_3_, Al_2_O_3_-SiO_2_-Ce_2_O_3_, and Al_2_O_3_-SiO_2_-Y_2_O_3_ at 1600 °C are summarized in Figure 4a [7,8,9,18,48]. It can be seen that the liquid regions of different system are roughly located in the same region. When the experimental results of Iftekhar et al. were introduced, only two experimental points of homogenous liquid phase are located outside the calculated liquid region. It may be caused by the deviation of the La_2_O_3_-SiO_2_ ternary system. Furthermore, other liquid phases and crystallized phases are located inside and outside of liquidus at 1600 °C, respectively. 

**Figure 5:**
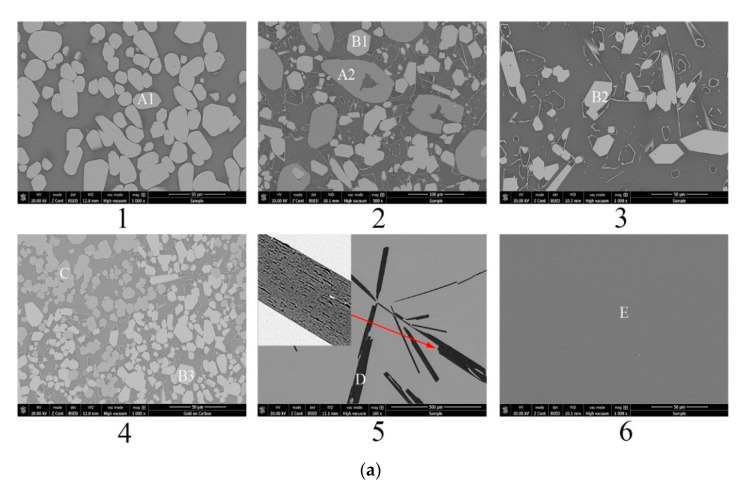

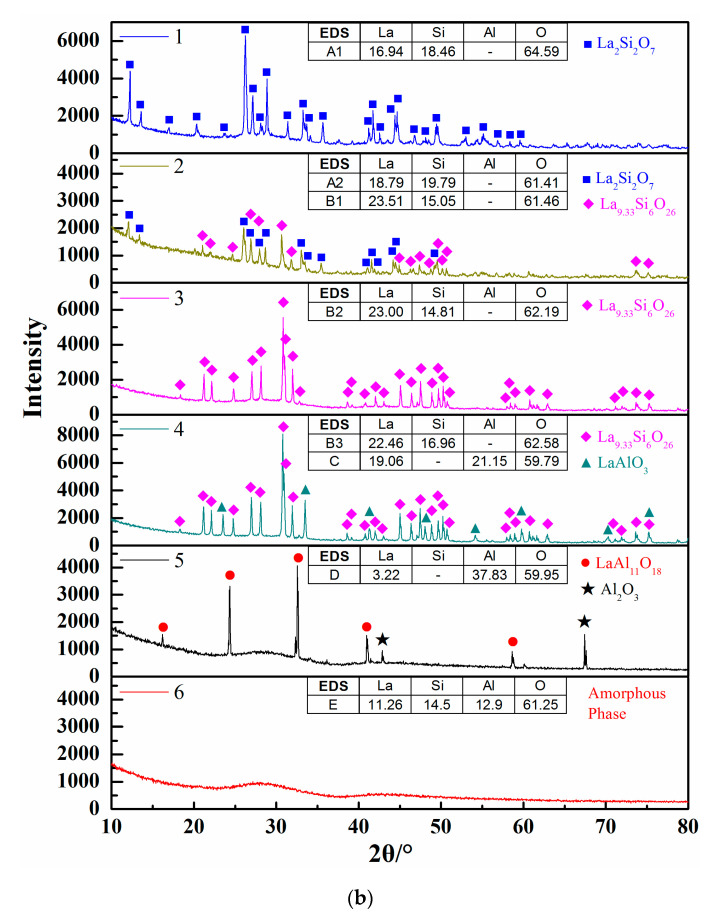
Morphology and component analysis of phases formed at 1500 °C. (**a**) SEM images of phases formed at 1500 °C; (**b**) XRD patterns of phases formed at 1500 °C, 1–6 represent the XRD patterns of experimental points 1-6 marked in Figure 4b, respectively.

Figure 4b shows the calculated isothermal section diagram at 1500 °C associating with the experimental results in the tube furnace. All the 18 prepared samples were observed and the symbols with the same shape indicate the same precipitated phase. For example, four solid squares located at the top left corner mean the precipitated phase analyzed by EDS and XRD is La_2_Si_2_O_7_. The micromorphology and phase compositions of quenched samples marked with circles in Figure 4b are given in Figure 5, and the elements ratio of EDS results of detecting points in Figure 5a, such as A1, B1, and D, are listed in Figure 5b. The compounds predicted from EDS results and XRD analysis are also given in Figure 5b. For the 18th sample (★ in Figure 4b), the peak of Al_2_O_3_ can be detected although the main phase is LaAl_11_O_18_. As the magnified image of the 5th picture shown in Figure 5a, some darker precipitates are present on the lighter phase. It was hard to distinguish the Al_2_O_3_ phase and LaAl_11_O_18_ phase from the EDS analysis because of the limited resolution of FE-SEM. However, both the Al_2_O_3_ and LaAl_11_O_18_ phases are detected from the XRD analysis. 

It can be seen from Figure 4b that the precipitates of experimental results are located in the corresponding primary crystal region of calculated phase diagram. The homogenous liquid phase points all locate within the region of liquidus at 1500 °C except for the rightmost point. It is easily concluded from Figure 5b that the precipitated phase in the La_2_O_3_-SiO_2_ system is not La_4_Si_3_O_12_ but La_4.67_Si_3_O_13_, which is recognized as La_9.33_Si_6_O_26_ in the XRD results.

**Figure 6:**
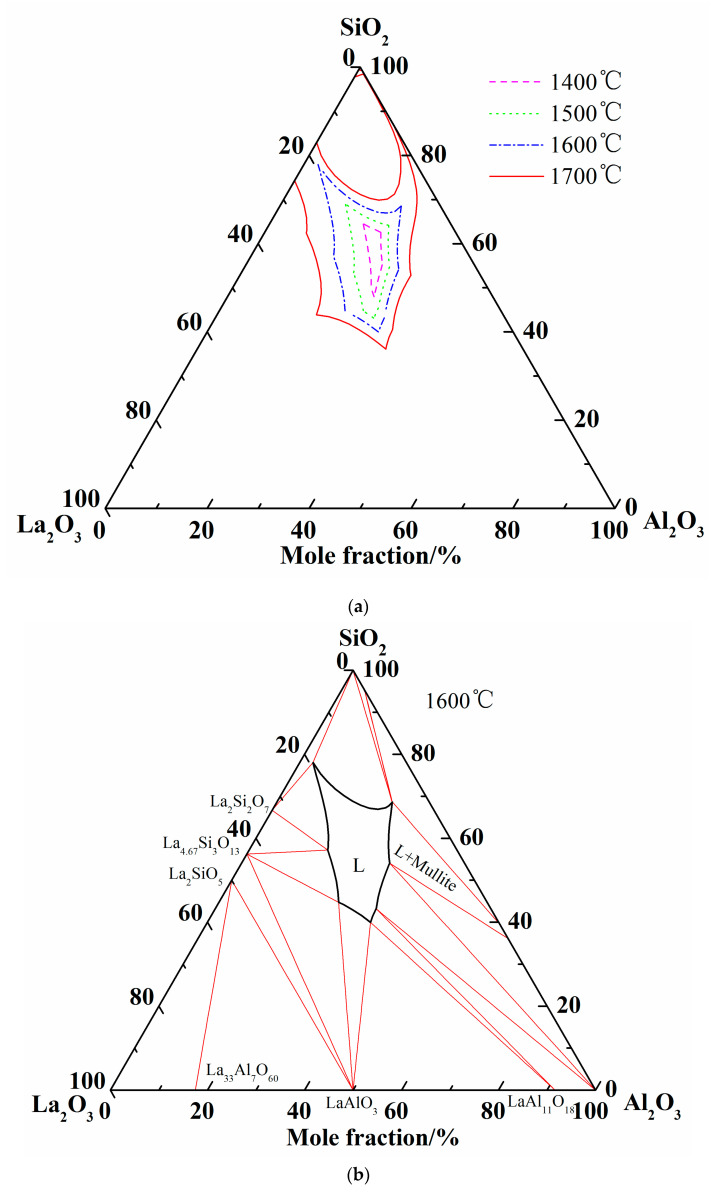

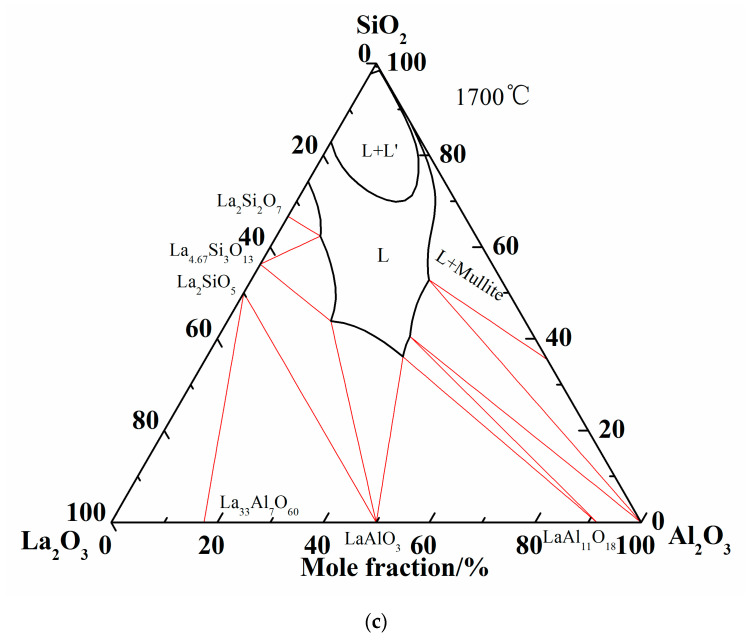
More calculations of Al_2_O_3_-SiO_2_-La_2_O_3_ ternary phase diagram. (**a**) Isothermal curves of Al_2_O_3_-SiO_2_-La_2_O_3_ system; (**b**) Isothermal section of Al_2_O_3_-SiO_2_-La_2_O_3_ system at 1600 °C; (**c**) Isothermal section of Al_2_O_3_-SiO_2_-La_2_O_3_ system at 1700 °C.

Summing up the above comparison of calculated phase diagram with the experimental results in former literature at 1600 °C and equilibrium experimental results at 1500 °C, it is obvious that the optimized thermodynamic parameters are reliable and the extrapolated Al_2_O_3_-SiO_2_-La_2_O_3_ phase diagram is reasonably precise. Therefore, the liquidus and isothermal sections of 1600 °C and 1700 °C are summarized in Figure 6. As shown in Figure 6c, two-liquid region (L + L’) is emerged in the isothermal section diagram of 1700 °C.

## 4. Summary

La_4.67_Si_3_O_13_ was first introduced to calculate the La_2_O_3_-SiO_2_ system by Redlich–Kister expression, and the Al_2_O_3_-SiO_2_ and La_2_O_3_-Al_2_O_3_ binary phase diagrams were also optimized by Redlich–Kister expression. The activities of Al_2_O_3_ and SiO_2_ were calculated in the Al_2_O_3_-SiO_2_ binary system, and then the Al_2_O_3_-SiO_2_-La_2_O_3_ ternary phase diagram was extrapolated by Kohler method. The conclusions are summarized as follows.

1. The liquid regions at 1500 °C and 1600 °C was verified by both experiments and data from publications. Comparing others’ work, the calculated ternary phase diagram is reliable and presents more precise.

2. It clearly indicated the compound in La_2_O_3_-SiO_2_ system is not La_4_Si_3_O_12_ but La_4.67_Si_3_O_13_. 

3. Based on the calculation results in the present study, a more reasonable model should be found to estimate the La_2_O_3_-SiO_2_ system in the future because of the deviation from experimental points. However, the ternary phases diagram in the present work present the relatively reliable region of liquid phase, and it is significant to the glass manufacture and other fields.

## Figures and Tables

**Table 1:** Thermodynamic properties of pure substances.

Formula	Phase	$H_{298}^{0}\;(k\cdot J\cdot {\mathrm{mol}}^{-1})$	$S_{298}^{0}\;(J\cdot {\mathrm{mol}}^{-1}\cdot K^{-1})$	*C_p_* = *A* + *B*·10^−3^ *T* + *C*·10^5^ *T*^−2^ + *D*·10^−6^ *T*^2^ (J·mol^−1^·K^−1^)				Applicable Temperature
				*A*	*B*	*C*	*D*	(K)
La_2_O_3_	S [21]	−1793.702	127.319	119.733	14.225	−13.506	-	298–2553
	L [22]	−1728.858	152.423	119.729	14.230	−13.500	-	2553–3000
Al_2_O_3_	S [21]	−1675.692	50.936	109.909	22.065	−33.284	−4.085	298–2327
	L [23]	−1596.533	43.569	192.464	-	-	-	2327–3000
SiO_2_	S [21]	−910.857	41.463	72.923	1.148	−41.848	0.036	298–1996
	L [21]	−906.604	51.029	85.772	-	-	-	1996–3000
LaAlO_3_	S [24]	−1794.300	85.936	111.010	13.481	−21.129	-	800–1500
LaAl_11_O_18_	S [22]	−10,107.591	383.296	697.843	103.054	−258.334	−19.660	298–2201
La_33_Al_7_O_60_	S [22]	−35,652.907	2404.150	2381.521	295.843	−382.856	−12.510	298–2161
La_2_Si_2_O_7_	S [25,26]	−3815.699	210.271	220.760	72.124	−41.832	−0.029	298–1000
La_2_SiO_5_	S *	−2853.310	168.782	182.9117	27.278	−33.6	-	298–3000
La_4.67_Si_3_O_13_	S *	−7302.500	421.467	463.165	73.192	−86.8	-	298–3000

* Derived from thermodynamic properties of La_2_O_3_ and SiO_2_ [27].

**Table 2:** Optimization parameters of the binary systems.

*L_i_* = *a* + *bT*	-	*L*_0_	*L*_1_	*L*_2_	*L*_3_
Al_2_O_3_-SiO_2_	*a*	19,570.28	14,875.48	5640.02	-
	*b*	−10.49	−0.71	1.21	-
La_2_O_3_-Al_2_O_3_	*a*	−129,195.34	−15,094.54	393,830.17	-
	*b*	−26.05	0.72	−176.58	-
La_2_O_3_-SiO_2_	*a*	−352,587.83	−119,125.74	513,748.94	435,926.54
	*b*	78.86	22.64	−211.27	−166.77
