# Correction: Li et al. Liquid Regions of Lanthanum-Bearing Aluminosilicates. *Materials* 2020, *13*, 450

**DOI:** 10.3390/ma17040940

**Published:** 2024-02-18

**Authors:** Yandong Li, Tongsheng Zhang, Yefeng Feng, Chengjun Liu, Maofa Jiang

**Affiliations:** 1 Key Laboratory of Extraordinary Bond Engineering and Advanced Materials Technology (EBEAM), Yangtze Normal University, Chongqing 408100, China; andyydlee@gmail.com (Y.L.); feng_ye_feng@126.com (Y.F.); 2 Key Laboratory of Ecological Utilization of Multi-Metallic Mineral of Education Ministry, Northeastern University, Shenyang 110819, China; liucj@smm.neu.edu.cn (C.L.); jiangmf@smm.neu.edu.cn (M.J.); 3 National Center for International Research of Clean Metallurgy, Central South University, Changsha 410083, China

In the original publication [1], there was a mistake in *Figure 2c* Phase diagram of La_2_O_3_-SiO_2_ system as published. The wrong figure was one progress picture based on the Toropov group’s older literature data, which is inconsistent with the full text description. The corrected *Figure 2c* for the phase diagram of the La_2_O_3_-SiO_2_ system appears below.

There was an error in the original publication. One chemical formula La_4_Si_3_O_12_ should be La_4.67_Si_3_O_13_ to be consistent with the paper. A correction has been made to *3.1.3. The La_2_O_3_-SiO_2_ System*:

The La_2_O_3_-SiO_2_ system is rarely studied by experiment or simulation, and different opinions on the intermediate phases have always been there. In 1961, Toropov released the La_2_O_3_-SiO_2_ phase diagram, and the intermediate compounds are La_2_Si_2_O_7_, La_2_SiO_5_, and La_4_Si_3_O_12_. In 1982, Bondar, from the same research group as Toropov, modified the La_4_Si_3_O_12_ phase to La_4.67_Si_3_O_13_. Li finished the calculated La_2_O_3_-SiO_2_ system employing simplified thermodynamic properties in 1999 [11]. However, the adoptive compound was La_4_Si_3_O_12_. Kim only calculated the two-liquid region using the Redlich–Kister expression [44]. However, the number and values of parameters given by Li and Kim are completely different. In this work, La_2_Si_2_O_7_, La_2_SiO_5_, and La_4.67_Si_3_O_13_ were chosen as the intermediate compounds, which were found in the equilibrium experimental results, as shown in Figure 5 later. Since the available experimental points are from Toropov and Bondar, the interaction energy of solution phase and the derived thermodynamic parameters of silicates were optimized. The calculated phase diagram is presented in Figure 2c [45,46]. It can be seen that most of the experiment points have good agreement with the calculation results.

The authors state that the scientific conclusions are unaffected. This correction was approved by the Academic Editor. The original publication has also been updated.

## Figures and Tables

**Figure 2:**
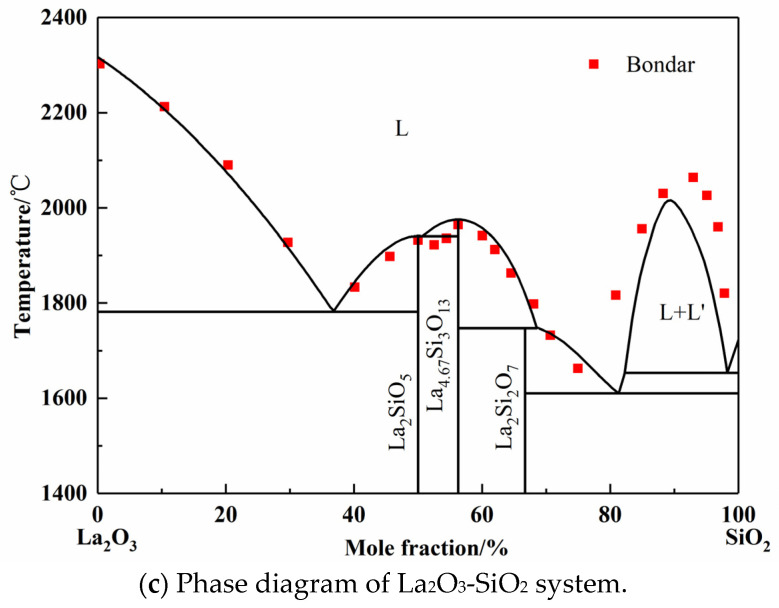
Calculated phase diagrams of binary systems.
